# A coach-assisted, online parenting programme to support parents of adolescents who refuse school: evidence of acceptability and feasibility

**DOI:** 10.1192/bjo.2025.61

**Published:** 2025-06-02

**Authors:** Anna Smout, Glenn Melvin, Mairead Cardamone-Breen, Anthony Jorm, Jue Xie, Tom Bartindale, Patrick Olivier, Joshua Seguin, Ling Wu, Marie B. H. Yap

**Affiliations:** Turner Institute for Brain and Mental Health, School of Psychological Sciences, Monash University, Melbourne, Australia; School of Psychology, Deakin University, Melbourne, Australia; Melbourne School of Population and Global Health, University of Melbourne, Melbourne, Australia; Action Lab, Department of Human-Centred Computing, Monash University, Melbourne, Australia

**Keywords:** School refusal, internalising disorders, parenting, adolescent, digital intervention

## Abstract

**Background:**

There is a need for developmentally tailored intervention approaches that empower parents to respond to adolescent school refusal in the context of internalising disorders. Partners in Parenting Plus-Education (PiP-Ed+) is a manualised coach-assisted online parenting programme that has been co-designed with parents, youth and education-sector experts to fill this gap. It addresses multiple parenting factors associated with adolescent school refusal and internalising disorders.

**Aims:**

This study aimed to evaluate the acceptability, feasibility and preliminary indications of efficacy of PiP-Ed+.

**Method:**

An open-label, uncontrolled trial was conducted using a mixed-methods design. Participants were 14 Australian parents of adolescents (12–18 years) who had refused school in the context of internalising disorders.

**Results:**

PiP-Ed+ was viewed as highly acceptable and feasible. Coaching sessions in particular were perceived as valuable and appropriate to the parents’ level of need, although longer-term support was suggested to sustain progress. Between baseline and post-intervention, there were significant increases in parents’ self-efficacy to respond to adolescent school refusal and internalising problems, and concordance with evidence-based parenting strategies to reduce adolescent anxiety and depression. Days of school refused and carer burden did not change.

**Conclusions:**

Findings support the value of proceeding to evaluate the efficacy of PiP-Ed+ in a randomised-controlled trial. Results are interpreted in the context of study limitations.

School refusal is a school-attendance problem that involves the experience of emotional distress at the prospect of attending or remaining at school, often resulting in school absence.^1^ School refusal presentations, particularly during adolescence, often co-occur with phobic anxiety, or combined anxiety and depressive disorders.^2–4^ The co-occurrence of school refusal and internalising disorders in this formative developmental period has been associated with limited treatment response in the medium and long term, prolonging impairment and complicating recovery.^5,6^

A range of interventions have been applied and evaluated in the treatment of school refusal, including psychodynamic approaches,^7^ family therapy^8,9^ and cognitive–behavioural therapy (CBT), with or without the addition of pharmacotherapy.^5,10–12^ Among them, CBT has received the most rigorous empirical attention and support.^13,14^ However, between one- and two-thirds of adolescents do not respond to this treatment modality,^15^ which underscores the need to explore effective alternatives or adjuncts to existing treatments.^12^

There is broad agreement about parents’ pivotal role in responding to school refusal, as reflected in the inclusion of parent components in all treatment protocols for adolescent school refusal to date.^15^ However, there remains a dearth of understanding about the role of parental involvement in school-refusal treatment outcomes. Modifiable parenting and family factors (e.g. parent psychopathology, parental self-efficacy (PSE), maternal overprotection, family functioning, parent–child conflict) have been empirically associated with school refusal, and overlap with those associated with adolescent internalising disorders.^16,17^ Despite this, there remains a gap in interventions for school refusal that seek to empower parents to support their adolescent, and the heterogeneous nature of school refusal means that parents may need individualised guidance to adequately respond.^18,19^ Because of common barriers to parents’ engagement with face-to-face interventions (e.g. scheduling, child care conflicts),^20,21^ or interventions requiring their adolescent’s participation, online parenting programmes that target adolescent school refusal hold great potential. The Partners in Parenting Plus-Education (PiP-Ed+) programme was developed to address this gap.

PiP-Ed+ is a newly developed adaptation of the Partners in Parenting Plus programme (PiP+ programme; formerly known as the Therapist-Assisted Online Parenting Strategies (TOPS) programme).^22^ PiP+ is a manualised coach-assisted online parenting intervention designed to improve evidence-based risk and protective factors for adolescent internalising disorders.^22,23^ PiP+ has been shown to be highly acceptable to parent and professional stakeholders,^19,22^ and associated with improvements in PSE, parent–adolescent attachment, family functioning and parent concordance with evidence-based parenting strategies to respond to adolescent anxiety and depression.^24^ PiP+ was therefore ideally placed for adaptation to address adolescent school refusal.^19^

The adaptation and development of PiP-Ed+ occurred over multiple phases. Adaptation activities included the following: (a) a Delphi expert consensus study (currently in preparation) to develop evidence-based, expert-endorsed parenting strategies for responding to school reluctance or refusal^25^; (b) qualitative interviews with parents from the original PiP+ trial, whose adolescent was struggling with school attendance during their participation,^19^ to identify adaptations required to better address their needs; and (c) co-design workshops with parents, educators and young people with professional or lived experience of school refusal^18^ to develop the adaptations to PiP+. The resulting PiP-Ed+ intervention is described in the Methods section.

## The present study

The Medical Research Council (MRC) recommends that complex health interventions are designed and evaluated in a phased, stepwise manner, with acceptability of the intervention established during the feasibility phase and before conducting large-scale clinical trials.^26^ In the development of health interventions, establishing intervention acceptability and feasibility can enhance uptake and effectiveness.^26,27^ Therefore, this study aimed to evaluate the acceptability, feasibility and preliminary indications of the efficacy of PiP-Ed+ among parents of adolescents who refuse school in the context of clinical-level anxiety or depression, in an open-label, uncontrolled trial. A mixed-methods approach was adopted for completeness,^28^ that is, to generate nuanced and comprehensive insights regarding programme acceptability and feasibility that can inform programme improvements.^29,30^ No directional hypotheses were specified because of the exploratory and mixed-methods nature of the study.

The specific aims are as follows.

- Develop a comprehensive understanding of acceptability through quantitative and qualitative examination.
- Examine programme feasibility by monitoring programme completion and adherence rates.
- Assess the intervention design’s validity from the perspective of parents. That is, what aspects of the programme *design* (e.g. online modules, coaching sessions) were perceived as factors that contributed to any observed improvements in PSE to respond to their adolescent’s difficulties.
- Gather preliminary indications of intervention efficacy, represented by quantitative change from pre- to post-intervention in (i) PSE to respond to adolescent school refusal, anxiety and depression, (ii) days of school refused (adolescent), (iii) concordance of parenting behaviours with evidence-based parenting guidelines^31^ and (iv) carer burden.

## Method

### Participants

Participants (*N* = 14) were mothers of adolescents aged 13–16 years who experienced school refusal. They were aged 36–54 years, and lived in Victoria, Australia. According to parents’ self-report, adolescents had missed 4.8 of the preceding 10 school days, on average. Sample characteristics are provided in Table 1. The sample size of 14 exceeded that recommended for pilot studies (*N* = 10).^32^

**Table 1:** Sample characteristics of baseline participants and their adolescents (*N* = 14)

Variable	Descriptor	
Parent characteristics		
Gender, *n*	Woman	14
Age in years, mean (range)		46.8 (36–54)
Ethnicity	Australian/New Zealand	11
	European – Eastern	1
	European – Western	1
	South Asian	1
Highest level of education, *n*	Higher education qualification	12
	Secondary qualification	1
	Part high-school completion	1
Relationship status, *n*	Single, separated or divorced	5
	Married/de facto	11
DASS-21, *n*	Mean (s.d.)	11.0 (6.4)
Symptom elevation (DASS-21), *n*	Anxiety	4
	Depression	6
	Stress	2
Adolescent characteristics (parent report)		
Gender, *n*	Boy/man	8
	Girl/woman	5
	Non-binary	1
Age in years, mean (range)		14.5 (13–16)
School grade, range		7–10
Days refused of past 10 school days	Mean (range)	4.8 (0–10)
	Missing, *n*	3
RCADS-P-25, *n*	Mean (s.d.)	23.0 (8.9)
Symptom elevation (RCADS-P-25, *n*)	Total anxiety	5
	Total depression	11
	Total anxiety and depression	9

DASS-21, Depression, Anxiety and Stress Scales-21 Item Version; RCADS-P-25, Revised Child Anxiety and Depression Scale-Parent Report, 25-item version.

Note: DASS-21 symptom elevation: Anxiety ≥ 4, Depression ≥ 5, Stress ≥ 8^36^; RCADS-P-25 symptom elevation: *T* Score ≥ 65.^38^

### Inclusion and exclusion criteria

Eligibility was based on parent report. Eligible participants were parents or guardians of an adolescent aged 12–18 who was experiencing school refusal in the context of depression or an anxiety disorder. Parents were excluded if their adolescent was experiencing a physical health condition that precluded school attendance, had a mental health condition that required immediate clinical prioritisation, had co-occurring autism spectrum disorder (ASD) or intellectual disability or who experienced the onset of school refusal before the 2022 school year. This was because the programme was not tailored for adolescents with these chronic/complex needs. Parents were required to live in Australia, be fluent in English and have capacity to attend regular online coaching sessions.

### Recruitment and procedures

This trial was approved by the Monash University Human Research Ethics Committee (MUHREC ID: 32704) and prospectively registered with the Australian New Zealand Clinical Trials Registry (ANZCTR; ACTRN12622000977774) on 11 July 2022. Participants were recruited in August 2022 through circulation of the study information and flyer by email to professional networks of the research team, primarily those working in well-being in educational settings. Interested participants completed an online expression-of-interest form to determine basic eligibility criteria. Potentially eligible parents then completed a screening phone call with a member of the research team to confirm eligibility and ascertain whether the programme was suitable for the needs of the family.

Eligible parents reviewed the participant explanatory statement and provided informed consent via the trial website (https://pip-ed.web.app/). Registration involved viewing a brief animated onboarding video, answering demographic questions about themselves and their adolescent and completing a baseline survey package (30–45 min). Immediately after submitting their baseline survey, parents received a tailored feedback report about their parenting strengths and areas for development based on their responses to the Parenting to Reduce Adolescent Depression and Anxiety Scale (PRADAS).^31^ Parents then confirmed which optional modules they would like to complete in addition to the five ‘required’ modules. After selecting their modules, parents had immediate access to their first module and were contacted to schedule their first coaching session. A module was required to be completed before attending the corresponding coaching session. Parents were encouraged to complete one module and coaching session per week, and had a maximum of 12 weeks to complete the six to eight modules with corresponding coaching sessions. Parents received automated email/SMS notifications when their next module became available, and SMS reminders for coaching sessions. Coaching sessions were run via video-conference.

At 90 days post-baseline, parents were alerted by email and SMS that their post-intervention survey was available. Parents received a maximum of two additional reminders if they had not completed the survey. At this time, parents were also invited to a 45-min qualitative interview to share their feedback about the programme, via Zoom. Parents received an online gift voucher for completion of the survey and interview (AUD$20 for each component; total AUD$40).

### The PiP-Ed+ intervention

The PiP-Ed+ programme aims to increase parents’ self-efficacy and alignment with evidence-based parenting recommendations to support their adolescent by providing personalised feedback about their current parenting, and equipping them with evidence-based parenting strategies to respond to school refusal, anxiety and depression. Although the programme is designed in a single-participant format, co-parents were encouraged to attend if they wished, with one parent nominated as the primary participant.

PiP strategies, including those relevant to school refusal, are based on parenting guidelines developed through systematic reviews of research evidence and Delphi studies of international expert consensus.^16,17,25,33,34^ The intervention was delivered online and consisted of up to 13 parent-led, online modules covering different topics relating to parenting an adolescent with school refusal and anxiety and/or depressive disorders (see Table 2 for an outline of module topics and content). Online modules consist of educational materials, case vignettes, reflective activities, video and audio clips, quizzes and goal-setting exercises, and each takes 20–35 min to complete. Online modules, completed weekly, were then supplemented by a maximum of eight one-on-one video-conferencing sessions with a PiP+ coach (registered or provisionally registered psychologists). Each 60-min coaching session followed a manualised structure including check-in, goal review, reiteration of key module content, a reflective activity and goal setting. Throughout, coaches supported parents in making changes in their parenting in adherence with the recommended strategies, and facilitated the individual tailoring of content to parents’ unique circumstances. Each week, parents selected a new topic-specific parenting goal from predetermined options presented in the corresponding online module to work towards (see Table 2).

**Table 2:** Overview of topics comprising Partners in Parenting Plus-Education (PiP-Ed+) and focus of each online module and coaching session

Topic name	Focus of online module content	Coaching session activity	Example parenting goal
Understanding anxiety and depression (required)	Delineating symptoms of anxiety and depression from typical adolescent developmental changes	Reflect on internalising symptoms experienced by the adolescent, and the impact of these on their socioemotional development.	Think about what factors in your teen’s life could be contributing to their depression or anxiety disorder. Have a think about what you might be able to do to reduce the impact of these factors on your teenager.
Connect (required)	Improving parent–child communication and relationships	Consider the thoughts and emotions behind behaviour/reactions in a challenging situation (such as a school morning) from both parent and adolescent perspectives.	Use ‘identify, validate & understand’ techniques in conversations with your teenager.
Breaking the anxiety cycle (required)	The impact of avoidance and parental accommodation on anxiety maintenance and how parents can support their adolescent to face their fears	Discuss the principles of graded exposure. Explore opportunities for parents to reduce accommodation of anxiety and model adaptive coping strategies.	Model helpful anxiety management strategies for your teen. The next time you are confronted with an anxiety-provoking situation, show your teen how you actively use strategies to deal with your own anxieties.
Understanding school refusal (required)	Understanding school refusal as an expression of distress that is likely underpinned by interacting individual, family and school factors	Consider school refusal like the visible part of an iceberg. Explore, and build a visual depiction of, factors below the surface that may be contributing to their school refusal.	If you haven’t spoken with your teen recently about going to school, set aside a time in the next week to have a conversation. Try to focus on understanding the current reasons for their school refusal rather than solving the problem for them.
Working together to overcome school refusal (required)	Practical tips for working together with their adolescent and school staff to overcome the problem, including key components of a return-to-school plan	Discern parents’ current priority regarding collaborating with others in support of their teen. Develop a related SMART goal.	With your teen, school staff, and any professionals involved, start developing or revising a return-to- school plan for your teen.
Calm versus conflict (optional)	How to manage and resolve conflict effectively, including remaining calm and using assertive communication skills	Reflect on parent and teen factors that can lead to/exacerbate conflict. Explore opportunities for parent to model effective conflict resolution strategies.	Practise using assertive communication with your teenager.
Raising good kids into great adults (optional)	Establishing and maintaining developmentally appropriate expectations and boundaries for behaviour	Explore opportunities for parent to create appropriate family rules. Discuss strategies for creating and upholding appropriate rules, including consequences.	Review your existing rules and consequences. See if any of your existing rules or consequences need to be adjusted to give your teen more freedom.
Maintaining the gains (required)	Principles of relapse prevention and how parents can support this	Consider parenting and self-help strategies that have been most helpful for teen, and possible warning signs for relapse. Reflect on parent experiences and progress with the programme.	Reflection: What are the main things you’ve got from the programme? This could be something you’ve learned, a new skill or strategy, a reflection or something you’ve discussed with your coach.
Partners in problem solving^a^	Supporting adolescents to develop effective problem-solving skills	N/A	Talk with your teen about a problem they are experiencing and work with them to apply the 6 problem-solving steps.
Nurture roots & inspire wings^a^	Maintaining parent–adolescent connection while promoting development of age-appropriate independence	N/A	Spend some one-on-one time with your teenager.
Good health habits for good mental health^a^	Supporting healthy habits related to sleep, diet, exercise, screen-time and substance use	N/A	Have a conversation with your teenager about the types of social media they use.
Good friends = supportive relationships^a^	Supporting adolescent development of social skills	N/A	Encourage your teen to do something kind for a friend or family member.
Parenting through the pandemic^a^	Responding to the challenges of the pandemic with confidence	N/A	Pick one self-care activity that you could do more of this week.

SMART, Specific, measurable, achievable, relevant and time-bound.

a. Optional online module offered without corresponding coaching session.

Two topics were newly developed for the PiP-Ed+ intervention: ‘Understanding school refusal’ and ‘Working together to overcome school refusal’. The remaining topics constituted the original PiP+ intervention.^22^ The core PiP-Ed+ intervention included six ‘required’ topics and two ‘optional’ topics, with coaching sessions offered for each (see Table 2). Parents had access to five additional online modules, offered without a corresponding coaching session. In addition, parents were provided with access to a collaborative coach-parent document housed on Google Slides, called the ‘Working Together Resource’. The resource was designed to support families and schools to work together in support of an adolescent struggling with school attendance, by guiding parents to gather and record information that is helpful to share between families and schools, with the support of their PiP-Ed+ coach. The questions included in the Working Together Resource were informed by the collective perspectives shared by parent, education-sector and youth lived-experience stakeholders during co-design workshops.^18^ Sample content from the online modules, coaching sessions and Working Together Resource are provided in Supplement A.

All PiP-Ed+ coaches completed standardised training in the delivery of the manualised programme. Coach training involved (a) self-led familiarisation with programme software and training and session manuals, (b) observation of pre-recorded example coaching sessions, (c) attending a live seminar (developed and led by A.S. in consultation with G.M.) about school-refusal case formulation, treatment principles and associated clinical challenges/considerations when working with parents in this context and (d) completion of a recorded mock coaching session followed by peer feedback. All PiP-Ed+ coaches engaged in weekly group supervision led by G.M., a registered clinical psychologist with extensive clinical expertise in school refusal, and M.B.H.Y., founder and lead researcher of the PiP programme with extensive expertise in parenting and youth mental health. Fidelity to the intervention was supported by detailed, structured coaching manuals, training procedures and weekly group supervision.

### Quantitative measures

#### Descriptive characteristics

*Demographic information.* At registration, parents provided basic demographic information for themselves and their child, including age, gender identity, ethnicity, education level and school grade.

*Parent mental health.* Symptoms of depression, anxiety and stress among parents were assessed using the 21-item version of the Depression, Anxiety, and Stress Scale (DASS-21).^35^ Subscale scores were categorised according to severity (from ‘normal’ to ‘extremely severe’) using the authors’ cut-offs.^36^ In the current study, Cronbach’s α = 0.84.

*Adolescent symptoms of anxiety and depression (parent report).* Adolescent symptoms of anxiety and depression were assessed using the parent-reported, 25-item version of the Revised Children’s Anxiety and Depression Scale (RCADS-P-25).^37^ Item scores are summed to yield three subscale scores (total anxiety, total depression and total anxiety and depression), and converted to normed *T* scores. *T* scores of 65–69 indicate the borderline clinical threshold, and scores of 70+ the clinical threshold.^38^ In the current study, Cronbach’s α = 0.87.

#### Acceptability

*Primary outcome: Client Satisfaction Questionnaire (CSQ-8).* The CSQ-8^39^ was used to assess parent satisfaction with the PiP-Ed+ intervention. In the current study, Cronbach’s α = 0.91. The CSQ-8 was administered 90 days after completion of baseline assessment (post-intervention). Scores of individual items in the CSQ-8 were summed to yield a global rating of programme satisfaction ranging from 8 to 32, where low acceptability = 8–20, medium = 21–26 and high = 27–32.^34^

#### Feasibility

*Programme completion rates* were measured as the percentage of *selected* online modules and coaching sessions that were *completed* by parents; that is, 100% × [(completed modules) / (selected modules)]. Only the eight topics comprising both an online module and corresponding coaching session in this trial (see Table 2) were included in the calculations. Percentages were calculated separately for online modules and coaching sessions. A completion rate of 75% or higher was considered feasible, as parent completion of five out of six (83%) required topics was determined as the minimum to retain intervention fidelity.

*Adherence to programme requirements* was measured as the percentage of participants who completed the six required online modules and corresponding coaching sessions. Percentages were calculated separately for online modules and coaching sessions, as follows: 100% × [(no. participants whose observed usage equals their intended usage) / (no. participants who received the intervention)], where observed usage refers to completed modules and coaching sessions, and intended usage refers to selected modules and coaching sessions. Completion of five out of six online modules, and five out of six corresponding PiP-Ed+ coaching sessions, was considered successful adherence.

#### Validity of the intervention design

*Design validation* refers to the process of evaluating whether, and how, a system or technology accomplishes its intended objectives and meets user needs through use of/engagement with the technology as intended.^40^ Design validity was assessed during semi-structured qualitative interviews with parents, at least 90 days post-baseline. Interview questions were designed to assess *how* programme design features (such as programme content, software and delivery mechanisms) influenced parents’ self-efficacy to respond to their adolescent’s school refusal, and otherwise met their needs. Parents’ suggestions about how the validity of the intervention design could be enhanced were also explored. Qualitative responses were corroborated with scores on the Parental Self-Efficacy Scale-School Refusal (PSES-SR) and the CSQ-8.

#### Indications of intervention efficacy

*PSE to respond to school refusal.* This was assessed using the total score on the PSES-SR. The PSES-SR consists of 15 items that assess a parent’s current level of confidence in responding to their adolescent’s school refusal, measured on a four-point Likert scale (ranging from 0 = ‘not at all confident’ to 3 = ‘very confident’). The items assess core strategies and approaches endorsed in the PiP-Ed+ content, such as ‘*How confident do you feel about your understanding of the reasons for your child’s school refusal?*’. Possible scores range from 0 to 45. This scale was specifically developed as an outcome measure for the PiP-Ed+ intervention. It has been validated in a separate study (currently in preparation) concurrent to this trial, which found the PSES-SR showed good internal consistency, and construct, convergent and divergent validity among community and help-seeking samples. In the current study, Cronbach’s α = 0.94 (baseline) to 0.95 (post-intervention).

*Days of school refused.* Days of school refused by the adolescent was measured by parent self-report. Parents responded to a single item: ‘In the 10 school days prior to [assessment date], how many days of school had [adolescent name] refused to attend? Please do not include absences due to illness, appointments, or COVID-19 related circumstances. Please respond as accurately as possible, including partial days missed. It may help to look at your calendar, diary, or online parent portal (if you have one) to prompt your memory’. The question was administered twice, first with reference to the ten school days before the baseline assessment date, and second with reference to the ten school days before the post-intervention assessment date.

*Parenting risk and protective factors for adolescent depression and anxiety.* Parental concordance with a set of evidence-based parenting guidelines^33^ was measured using the PRADAS.^31^ The PRADAS is a criterion-referenced scale that assesses parental concordance with the guidelines in eight domains of parenting. Each domain/subscale (e.g. ‘parent–child relationship’) maps onto evidence-based risk and protective factors for adolescent depression and anxiety disorders that parents can potentially modify (e.g. parental warmth, aversiveness). Each of the 73 items is scored as concordant (1) or not concordant (0) with the guidelines to give a total score out of 73. In the current study, agreement coefficients^41^ were 0.97 (baseline) and 0.92 (post-intervention).

*PSE to reduce the risk of adolescent depression and anxiety.* PSE was measured using the Parental Self-Efficacy Scale (PSES).^42^ It consists of eight items measuring parent confidence in preventive parenting measured on a four-point Likert scale, with items summed to give a score range from 8 to 32 (from 1 = ‘not at all confident’ to 4 = ‘very confident’). In the current study, Cronbach’s α = 0.71 (baseline) to 0.90 (post-intervention).

*Carer burden.* Change in carer burden was measured using the Burden Assessment Scale (BAS).^43^ The BAS comprises 19 items assessing the extent to which parents experience observable and subjective consequences related to caring for their adolescent (e.g. financial problems). Items are scored on a four-point Likert scale (from 1 = ‘not at all’ to 4 = ‘a lot’) and summed to obtain an overall score ranging from 19 to 76, with higher scores indicative of higher experienced burden. In the current study, Cronbach’s α = 0.89 (baseline) to 0.97 (post-intervention).

### Qualitative measures

#### Programme acceptability, feasibility anddesign validity (qualitative interview)

Programme acceptability, feasibility and design validity were also assessed qualitatively via semi-structured interviews with parents, at least 90-days post-baseline. The interview schedule (Supplement B) was primarily based on items included in the Theoretical Framework of Acceptability (TFA) questionnaire,^44^ each of which assesses a theory-informed domain of programme acceptability.^27^ Open questions addressing feasibility of programme completion and design validity were also included.

### Data collection and analysis

#### Quantitative data

Quantitative analyses were conducted in SPSS Version 29 for macOS (IBM, Armonk, NY, USA; see https://www.ibm.com/products/spss-statistic). Scores on the CSQ-8 at post-intervention were analysed descriptively, and quantitative feasibility data were calculated as percentages. Paired-sample *t*-tests were conducted to assess pre-to-post change in PSE, parenting behaviours and carer burden, and adolescent school-refusal rates. We followed Cohen’s^45^ guidelines for interpreting mean effect sizes whereby *d* values of 0.2, 0.5 and 0.8 represent small, medium and large effect sizes, respectively.

#### Qualitative and mixed-methods data

Qualitative interviews were conducted one-on-one via Zoom video-conferencing software by a researcher who was not the parent’s coach. Parents were asked to share their perspectives about what they liked and disliked about the programme, how (if at all) the programme increased their confidence to respond to their adolescents’ difficulties and how the programme could be improved. To aid the interviewee, interview questions were read verbally and provided visually on PowerPoint slides. Interviews lasted between 36 and 57 min, were audio- and video-recorded and transcribed by a third party. Interview data were analysed thematically with themes identified inductively. Coding and analysis were led by the first author adhering to Braun and Clarke’s^46^ six-phase, reflexive application^46,47^ using NVivo 11 for macOS (Lumivero, Denver, CO, USA; see https://lumivero.com/product/nvivo/). All authors contributed to the review and refinement of initial themes. Material was only excluded if it was not related to intervention acceptability or validity, or was inaudible. Data collection stopped when all parents who had agreed to partake in the interview had done so.

Mixed-methods analyses were conducted using a concurrent, convergent approach in which quantitative and qualitative data were collected and analysed during the same phase of research, before merging results to create a corroborated interpretation.^48,49^ Joint displays^29^ were used to merge quantitative and qualitative data related to intervention acceptability, feasibility and validity. In the joint display, descriptive statistics and statistical analysis of survey data were consolidated with qualitative themes and sub-themes from interview analysis to create corresponding meta-inferences.

## Results

**Fig. 1:**
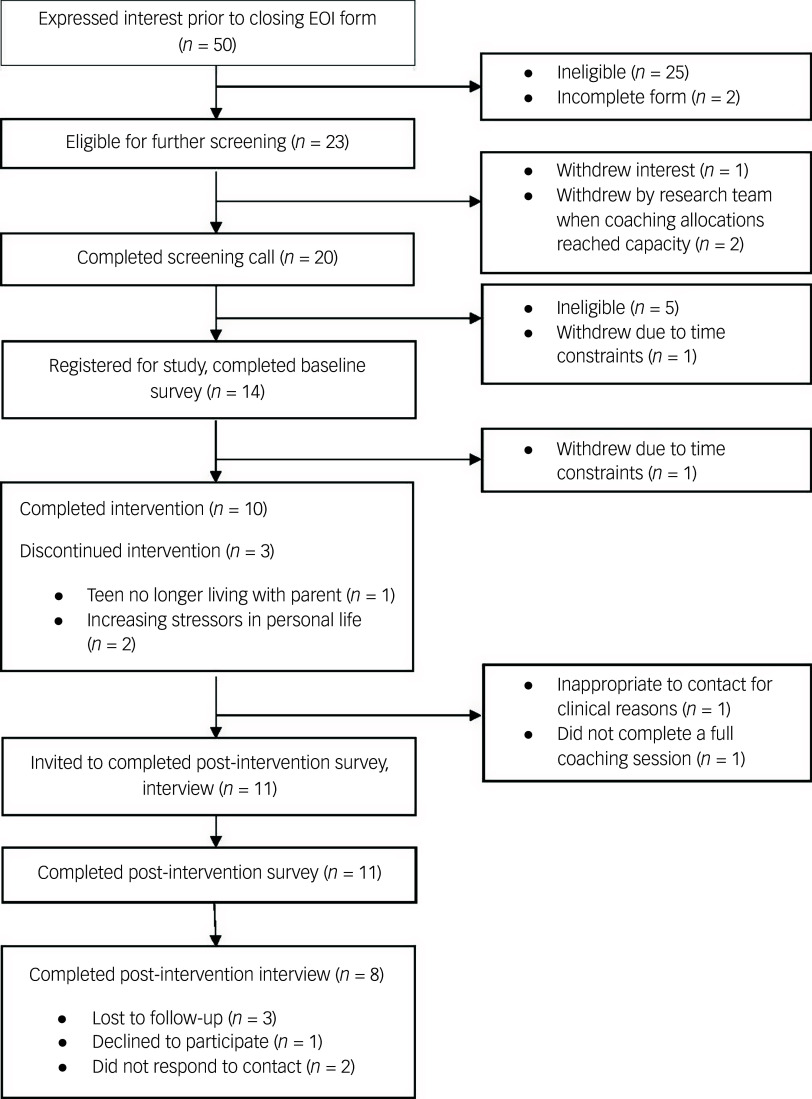
Participant flow diagram. EOI, expression of interest.

Parent and adolescent demographic characteristics are provided in Table 2, and the participant flow diagram is presented in Fig. 1. One participant opted to complete the programme with their co-parent as a dyad throughout. Of the 14 parents who registered to take part between 19 August and 21 September, 2022, all completed the baseline assessment, 11 completed the post-intervention survey and eight completed the post-intervention interview. Post-intervention survey data were collected between 1 December 2022 and 3 January 2023, and interviews were conducted between 5 December 2022 and 17 February 2023.

### Quantitative findings

Quantitative results are presented in Tables 3 and 4.

**Table 3:** Quantitative outcomes: programme acceptability and feasibility (*N* = 11)

Outcome	Descriptor	
90-day post-intervention survey, *n*	Complete	11
	Missing	3
Acceptability		
CSQ-8 Score, mean (s.d.)	Total score	30.6 (2.2)
Feasibility		
Programme completion rates, *n* (%)	Modules	11 (79%)
	Coaching sessions	10 (71%)
Adherence to programme requirements, *n* (%)	Modules	11 (79%)
	Coaching sessions	10 (71%)

CSQ-8, Client Satisfaction Questionnaire.

**Table 4:** Quantitative outcomes: preliminary indications of efficacy (*N* = 11)

Outcome	Descriptor				*n*		
90-day post-intervention survey	Complete				11		
	Missing				3		

Parenting self-efficacy; school refusal	Mean (baseline)	Mean (post-intervention)	Mean difference	s.d.	*t*	*p*	*d*
PSES-SR scores	19.7	37.8	−18.1	12.1	−5.0	<0.01*	1.9
School refusal							
Change in days refused in preceding 10 school days	4.8	3.8	1.0	3.8	0.9	0.4	0.3
Parenting to reduce adolescent depression and anxiety							
PRADAS scores	48.7	56.6	−7.9	4.6	−5.8	<0.01*	1.6
Carer burden							
BAS scores	47.4	47.9	−0.5	8.7	−0.2	0.8	0.1
Parenting self-efficacy; anxiety and depression							
PSES scores	23	31.8	8.8	3.8	−6.6	<0.01*	1.7

PSES-SR, Parental Self-Efficacy Scale-School Refusal; PRADAS, Parenting to Reduce Adolescent Depression and Anxiety Scale; BAS, Burden Assessment Scale; PSES, Parental Self-Efficacy Scale.

* *p* < 0.001; *d* = Cohen’s *d*.

#### Programme acceptability

The mean score on the CSQ-8 was 30.6 out of a possible 32 (s.d. = 2.2), indicating a high degree of acceptability.

#### Programme feasibility

*Programme completion rates.* Some 79% of parents (11 of 14) completed at least 75% of their selected modules and 71% of parents (10 of 14) completed at least 75% of their selected coaching-based components of the programme.

*Programme adherence rates.* Some 79% of parents (11 of 14) completed the six ‘required’ online modules and 71% of parents (10 of 14) completed the six corresponding coaching sessions.

#### Preliminary indications of efficacy

*PSES-SR.* Parenting self-efficacy to respond to adolescent school refusal significantly increased between baseline (M = 19.7, s.d. = 10.7) and post-intervention (M = 37.8, s.d. = 7.7), *t* = −5.0, *p* < 0.01. The effect size was large (*d* = 1.9).

**Fig. 2:**
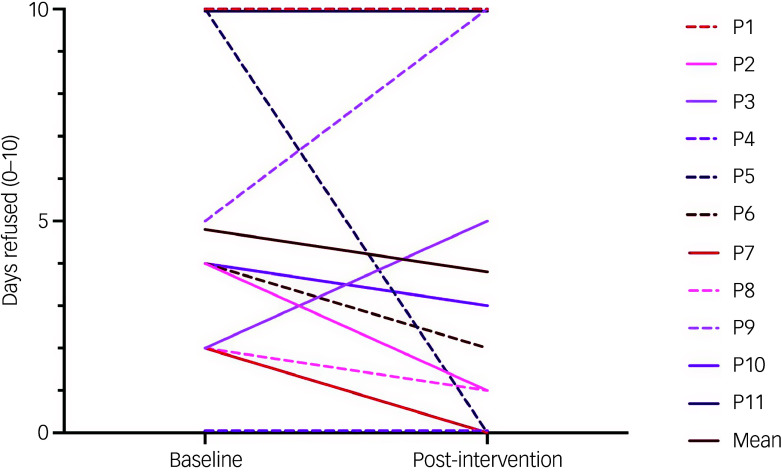
Days of school refused at baseline and post-intervention (*n* = 11). Each participant is represented by one line.

*School refusal.* There was no statistically significant change in days of school refused between baseline (M = 4.8, s.d. = 3.6) and post-intervention (M = 3.8, s.d. = 4.2), *t* = 0.9, *p* = 0.4. As can be observed in Fig. 2, compared to baseline, six adolescents refused fewer days of school at post-intervention, two adolescents refused more days and three adolescents refused the same number of days. Those who refused the same number of days refused zero days (*n* = 1) or 10 days (*n* = 2).

*PRADAS.* Scores on the PRADAS increased significantly between baseline (M = 48.7, s.d. = 5.0) and post-intervention (M = 56.6, s.d. = 5.2), *t* = −5.9, *p* < 0.01. The effect size was large (*d* = 1.6).

*BAS.* BAS scores did not change significantly between baseline (M = 47.4, s.d. = 11.2) and post-intervention (M = 47.9, s.d. = 11.4), *t* = −0.2, *p* = 0.8. To better understand this finding, patterns of associations among carer burden scores, baseline RCADS scores, days of school refused and PSE were explored post hoc as bivariate correlations. Carer burden scores at baseline and post-intervention were not significantly associated with any of these variables at either time point.

*PSES.* Scores on the PSES increased significantly between baseline (M = 23.0, s.d. = 4.0) and post-intervention (M = 31.8, s.d. = 4.5), *t* = −6.6, *p* < 0.01. The effect size was large (*d* = 1.7).

### Qualitative findings

Four overarching themes and 21 sub-themes (italicised below) were identified from the qualitative interviews conducted with eight parents. A description of the response patterns that exemplify each sub-theme is provided below, and representative quotes are provided in Table 5.

**Table 5:** Qualitative interview themes, sub-themes and representative quotes (*N* = 8)

Theme	Sub-theme	Quotes
Valued aspects	Coaching sessions were the most valuable part of the programme	’I think when you read through information, it’s all very logical and it’s not stuff that I don’t know but I think speaking with [Coach] makes it actually more, relatable and more specific to you. So I think that the programme by itself, without the coaching sessions, I don’t think it would work as well’. [P8]
	Qualities of their coach	’I think the skill of the person running the counselling sessions is really important I think, yeah there needs to be a level of maturity and a level of skill that’s really important and I think, [Coach] definitely had that’. [P6]
	Content was appropriate, engaging, comprehensive and helpful	’I liked how it [video example of inadvertent parent accommodation of adolescent anxiety] showed the mother’s approach… because that was a revelation to me that that’s what I always do… So seeing that and then seeing the other approach, in contrast, yeah, was like a revelation’. [P5]
	Experiencing positive outcomes	’I am way more confident… after this programme I actually understand what I was doing intuitively which was correct, but it made me more confident to actually understand what I’m doing and why I’m doing it’. [P4] ’We got [Child 5] back to school by the end of it… from complete non-attendance to complete attendance all the time. Not even a modified timetable’. [P5]
	Programme instilled a positive sense of accountability	’She [Coach] was relying on me to be there and I was relying on myself to be there and then I was like, right, I’ve got to put my mind into what we’re doing here’. [P3]
	Number and frequency of online and coached components	’I think the number is right. I think if you went much less than that you just wouldn’t actually get into enough of the nitty gritty, I think. I think any more than that, you know, how much more benefit, like what other modules could you add in’. [P6]
	Time commitment required to complete weekly activities made completing the programme achievable	’I just slotted it into my calendar and was able to fit it around my work… and family’. [P1]
	Programme was worth the time and effort	’It’s an effort – you’ve got to make yourself emotionally and mentally available to it… but I think it needs to be that to be beneficial’. [P6]
	Programme being free of charge	’The fact that this was a free programme was amazing for us because it gave me support which I normally would not get’. [P2]
	Flexibility of coaching sessions	’The programme was extremely flexible. If I couldn’t make it on a day, or whatever, [coach] was ready to postpone… So it worked well for me’. [P7]
	The timeliness of the programme	’I found that I was blaming myself for what was happening, and I knew that wasn’t really doing me any good so I was looking for the answers when this popped up and so therefore, this came at a really good time’. [P7]
Factors that contributed to increased parent confidence in responding to school refusal	The content (evidence-based parenting strategies)	’It’s made me realise that the things I was doing was right and even though everything doesn’t work straight away, it was just a really good reminder and refresher that I wasn’t alone, and the steps I was taking were to help him even if they were taking longer than I thought’. [P1]
	Encouragement and reassurance from their coach	’[Coach] was able to… make me see that even trying is an accomplishment. And also that there were little wins within that, even though you didn’t get… the outcome’. [P3]
	Coach tailoring of goal-setting activities	’We were applying it to our situation and discussing how we could have done it differently, how we could do it differently next time, so it was more like, hands on kind of thing’. [P4]
	Their own attitude and need	’Yeah. Because you can be easily defensive and say well I’m doing that. But then I’ll be like, I’ve done that but, you know we’re going to try it in a different way or try it again because I’m in a different mindset’. [P3]
Disliked aspects	Mismatches between some content and parent circumstances, needs or preferences	’Some of the suggestions of parenting are just so far ahead of where we are with our communication with our child’. [P6]
	Difficult for two parents to co-participate	’I had a couple of sessions with [coach de-identified] without [parent 16] and I enjoyed that and he wasn’t able to have a session with her on her own because it had to be with – I was told it had to be with the [primary] participant’. [P6]
	Some components were difficult to access or complete	’I did have trouble, having a go at the goals formally… I got busy and I forgot… I probably didn’t like the setting of the goals because I found it hard to achieve them, formally’. [P2]
	Issues related to video-conferencing	P5: ’That’s feedback that might be nice to tell people at the start… That, maybe this will bring out some emotion in you or something… Because then I wouldn’t have tried to smother it’. Interviewer: ’Mm. What do you think made you feel you needed to do that?’ P5: ’Just Zoom, I guess’.
Suggestions for improvement	Provide more support to sustain progress long term	’It’d be good to meet even six weeks later on with the same person, just to reflect on the programme and see what changes, what you’re able to adapt or if things are still the same… So not just after having a week to put things in practice’. [P1]
	Changes or additions to content and functionalities	’I think it’s probably having a bit of a choose your own adventure where if you’re coming to the end of your sessions but you actually haven’t made a super amount of progress you do a different module than the maintaining the gains people’. [P6]
	Involvement of co-parents, their adolescent and school staff	’I guess because part of the solution is also getting the school involved. Maybe running I mean some face-to-face sessions with the teachers there, and try and share that this sort of thing is happening, it’s real’. [P7]

#### Theme 1: valued aspects

Overall, participants described having a positive experience with PiP-Ed+. They highlighted that *coaching sessions were the most valuable part of the programme,* due in large part to the tailoring of programme content during coaching sessions. In addition, they valued the *qualities of their coach,* and felt the *programme content was appropriate, engaging, comprehensive and helpful.* In particular, they favoured video and audio clips included throughout modules, and the concise manner in which information was presented. Parents also reported that both they and their adolescent *experienced positive outcomes,* including increases in their parenting confidence, gaining a better understanding of their adolescent’s experience and improvements in their adolescent’s school attendance.

Parents also reflected on the ways in which the *programme instilled a positive sense of accountability,* whereby coaching sessions and goal-setting activities helped them to make time to think critically about their parenting, and translate their learning into action. They noted that the *number and frequency of online and coached components* was appropriate, the *time commitment required to complete weekly activities* was achievable and *the programme was worth the time and effort* because it was meeting their needs and providing desired results. Parents further explained that the *programme being free of charge* and the *flexibility of coaching sessions* made the programme feasible and accessible to complete. Finally, some parents commented on *the timeliness of the programme* relative to their circumstances.

#### Theme 2: factors that contributed to increased parent confidence in responding to school refusal

Parents reflected on numerous processes through which the programme design contributed to increases in their parenting confidence. Overall, the combination of the reputable *content (evidence-based parenting strategies), encouragement and reassurance from their coach* and *coach tailoring of goal-setting activities* appeared to give parents the confidence to try new things (translate learning into action) and persevere with learned strategies. Notably, many also believed that *their own attitude and need* affected programme completion and outcomes. That is, they believed their openness to receive and adopt feedback on their parenting, and immediate need for the support, contributed to the benefits and increases in confidence they experienced.

#### Theme 3: disliked aspects

Despite low consensus across participants regarding aspects of the programme they disliked, four were identified overall. First, there were *mismatches between content and the circumstances, needs or preferences of some parents.* For example, the Working Together Resource was seemingly irrelevant to the majority of parents. Reasons for this varied substantially between participants, and included the imagery being too young, already having a good relationship with school staff and the resource feeling too formal. Second, some parents found the programme’s focus on engaging one parent made it *difficult for two parents to co-participate.* Third, parents explained *some components were difficult to access or complete,* such as the Working Together Resource, which was housed on Google Slides and not accessible through the main PiP-Ed+ programme dashboard. Fourth, a small subset of parents highlighted *issues related to video-conferencing,* such as having difficulty finding a private space from which to attend.

#### Theme 4: suggestions for improvement

Suggestions for programme improvements varied in their degree of consensus among parents. There was highest agreement that future iterations of the programme should ideally *provide more support to sustain progress long term,* for example, through booster sessions or the inclusion of strategies for sustaining progress in programme content. Parents also suggested various *changes or additions to content and functionalities.* These included (a) offering an alternative to the final programme topic on relapse prevention (‘maintaining the gains’) for those who perceived their adolescent to have made little progress during their participation and (b) SMS reminders about goal selection and completion. Finally, a subset of parents advocated for greater *involvement of co-parents, their adolescent and school staff* in the programme. The one parent dyad who completed the programme together explained that functionalities which enabled parents to log in from separate devices and choose different weekly goals would have facilitated their joint participation.

### Mixed-methods findings

Mixed-methods findings were developed by merging quantitative and qualitative results, using a joint display (Supplement C). Themes and sub-themes arising from interviews mapped intuitively onto the corresponding quantitative outcomes. Regarding acceptability, the majority of sub-themes comprising ‘valued aspects’ confirmed and added context to the high CSQ-8 scores observed. Sub-themes within ‘disliked aspects’ and ‘suggestions for improvement’ then provided practical opportunities and suggestions for improving acceptability. Regarding the validity of the intervention design, the PiP-Ed+ content, coaching support and goal-setting activities combined seemingly contributed to increases in PSES-SR scores between baseline and post-intervention. Regarding feasibility, programme completion and adherence rates suggest the module-based programme requirements were slightly more feasible to complete than coaching-based requirements. Those who completed the whole programme (all interviewees) stated the flexibility of coaching sessions, no cost and appropriate time commitment had enabled their completion. Finally, one qualitative elaboration (parenting factors or circumstances that affected programme acceptability, feasibility and validity) was discerned by conducting the joint display. Specifically, the timeliness of the programme for parents and their own attitudes about/need for support were factors that related to the study aims but fell outside the direct influence of intervention design.

## Discussion

This study demonstrated the acceptability, feasibility and design validity of the PiP-Ed+ intervention. It also found statistically significant increases in (a) PSE to respond to adolescent school refusal and (b) anxiety and depression, and (c) concordance with evidence-based parenting strategies to reduce adolescent anxiety and depression. There was no statistically significant reduction in days of school refused or degree of carer burden between baseline and post-intervention. To our knowledge, PiP-Ed+ is the first programme designed to address parenting factors associated with adolescent school refusal developed primarily for, and with, parents. The good fit between PiP-Ed+ and the needs of the parent population included in the present study is likely because of the involvement of intended end-users throughout programme development and adaptation.^18,19,22^ High degrees of satisfaction with PiP-Ed+ resulted in few salient suggestions to further enhance acceptability, feasibility or design validity. The clearest opportunity for improvement was the provision of additional support to sustain progress long term. Parents requested booster sessions and the addition of strategies to maintain the adaptive parenting practices they had developed within programme content.

The inclusion of such maintenance strategies, such as booster sessions, in parenting programmes is common.^50^ Although empirical examination of discrete or incremental benefits attributable to maintenance strategies is limited,^51^ there is little evidence to support their efficacy.^52^ Paucity of evidence aside, parents’ knowledge that they have learnt specific skills to maintain their gains or will have ongoing access to support may augment their confidence upon finishing the intervention. In this context, digital interventions are ideally placed to offer long-term support features that circumvent the resource restrictions associated with long-term human delivery. For example, parents could opt into receiving automated reminders about programme content they found useful and prompts to develop and work towards parenting goals, or reflect on key challenges and progress, via email and/or SMS. Further research would be required to determine what features add value for participants and intervention outcomes. In the immediate term, other adaptations can be made to the programme in line with parent feedback. These include better tailoring of the features of the Working Together Resource to the adolescent cohort and improved facilitation of the involvement of co-parents in the intervention. The latter could be facilitated by enabling co-parents to create individual programme accounts to access their own personalised online module package, select and work towards different goals and, resources permitting, offering individual and combined sessions with their coach as required. Other points of feedback will not be translated into immediate adaptations because of low agreement between participants, but will be monitored in future trials.

Participants who attended the interview (the majority of whom were employed and had a parenting partner) endorsed the feasibility of completing the programme as intended. Ten out of 14 (71%) of the participants completed the full intervention after registration. Two participants who discontinued after commencing coaching were both separated/single parents of adolescents with clinically elevated depression and anxiety scores. Although other parents completed the intervention under these conditions, those who discontinued reported significant/unexpected changes in personal circumstances after programme commencement (e.g. involvement of child protective services). In summary, the programme shows promise in being feasible to complete, including among single, working parents and those whose adolescents were experiencing severe symptoms of anxiety and/or depression and were not attending school at all. Allowing for more flexibility in programme completion deadlines to cater to the needs of parents who experience an unexpected stressor or significant change in availability during programme engagement is an important consideration for future implementations.

Taken together, the findings support the validity of the intervention design, showing that the reputable, relevant information, paired with tailoring of content and beneficial therapeutic processes arising from the parent–coach relationship increased parents’ confidence in their capacity to respond to their adolescents’ difficulties. According to parents, these factors, in addition to positive experiences of accountability instilled by structured goal-setting activities supported by PiP-Ed+ coaches, were key mechanisms that produced behaviour change. Their reflections mirror principles (e.g. tailoring, praise, expertise, similarity) underpinning the Supportive Accountability Model^53^ and Persuasive Systems Design,^54^ both of which informed the design of PiP-Ed+. Indicators of intervention efficacy further affirmed the validity of the intervention design through significant increases in parent-reported self-efficacy and concordance with evidence-based parenting strategies, as related to adolescent school refusal, anxiety and depression.

As previously mentioned, there was no statistically significant reduction in days of school refused between baseline and post-intervention. It is worth noting that the majority of adolescents (*n* = 6, 55%) refused fewer days of school at post-intervention compared to baseline (two adolescents refused more days and three refused the same number of days). On the one hand, this finding is consistent with previous efforts to address adolescent school refusal that produce improvements, but not to aspired levels.^10,12^ On the other hand, this finding is promising, particularly in light of the fact the intervention was delivered only to parents and in the final term of the 2022 school year. The lack of significant effect perhaps reflects the fact that there are multiple drivers of school refusal, and parenting is just one. It is also worth highlighting that of the three adolescents who refused the same number of days, one had refused zero days on both occasions, and thus could not ‘improve’. The remaining two adolescents had refused ten school days at both timepoints. For these adolescents, it is somewhat unsurprising that a parent-only intervention did not culminate in substantial improvements in the short term, given the severity of their refusal. Taken together, these findings could conceivably reflect some early signs of improvement among adolescents with mild to moderate school refusal, with the acknowledgement that any significant indirect effects of the programme on adolescent outcomes would take longer to manifest.^55^ Moreover, if any indirect effect sizes were small, the current study would have been underpowered to detect them. Thus, the potential of the intervention to improve adolescent school attendance, particularly among adolescents with mild to moderate school refusal, should not be discounted without longer follow-up of adolescent outcomes.

Finally, consistent with outcomes of the PiP+ pilot evaluation,^24^ carer burden scores did not change between baseline and post-intervention. Supporting a child’s return to school requires substantial, daily parental effort and involvement. It is therefore conceivable that the degree of burden a parent experiences may increase or sustain until their child is attending without issue. Thus, even among parents of adolescents who had refused less school days at 90-day follow-up compared to baseline, limited change in carer burden between baseline and post-intervention is perhaps unsurprising. Future research will be required to further clarify these findings.

The results of this study should be interpreted with consideration of its limitations. First, the parents who did not complete the full programme as intended also declined participation in the evaluation interview. As such, available qualitative acceptability, feasibility and validity data may present an incomplete picture of the participant experience. Second, although the measures used have been validated in similar populations, all outcomes (except completion/adherence rates) were measured according to parent report, and are thus amenable to self-report biases. In particular, reasons for drop-out provided by parents may have been vulnerable to social desirability bias. Third, days of school refused were measured in whole days. Partial improvement in days attended was therefore not captured. Fourth, all participants in the present study were mothers and most had graduate/postgraduate qualifications and ethnically identified as Australian/New Zealander, limiting generalisability. This observation is not uncommon in the interventional parenting literature, and highlights the importance of tailoring recruitment approaches to engage a more representative sample of parents in future evaluations of PiP programmes. Finally, the small sample size and lack of control group preclude any conclusions about the efficacy of the programme.

The present findings support the acceptability, feasibility, design validity and preliminary indications of efficacy of the PiP-Ed+ programme and the value of proceeding to evaluate its efficacy in an appropriately powered randomised-controlled trial. The intervention addresses an important gap in support for parents of adolescents struggling with school refusal, anxiety and depression. Although parenting factors represent just one facet among a complex array of individual, school and community-level factors associated with school refusal,^56^ the present findings support the value of empowering parents in addressing adolescent school refusal.

## Supporting information

Smout et al. supplementary material Smout et al. supplementary material

## Data Availability

The data that support the findings of this study are available on reasonable request from the corresponding author, M.B.H.Y. The data are not publicly available as they contain information that could compromise the privacy of research participants.

## Supplementary material

The supplementary material can be found online at https://doi.org/10.1192/bjo.2025.61
