# Supplementary material for: A coach-assisted, online parenting programme to support parents of adolescents who refuse school: evidence of acceptability and feasibility
Source: BJPsych Open. 2025 Jun 2;11(4):e115. doi: 10.1192/bjo.2025.61 (PMC12188227; doi:10.1192/bjo.2025.61)
Supplement: Smout et al. supplementary material [file S2056472425000614sup001.docx]

## **Supplement A: Sample content from the online modules, coaching sessions, and Working Together Resource**

In combination, the following content and activities were designed to empower parents to:

- Develop and consolidate their understanding of what underlies and maintains their adolescent’s school refusal difficulties.
- Build a shared understanding of this with their PiP-Ed+ coach, and identify areas worthy of further exploration.
- Consider that there may be multiple reasons for a teen’s school refusal (many of which are not related to their parenting) and how they each interact.

| Online module content | Coaching session activity | Working together resource excerpt |
| --- | --- | --- |
| The iceberg metaphor is introduced in the online module ‘Understanding School Refusal’.  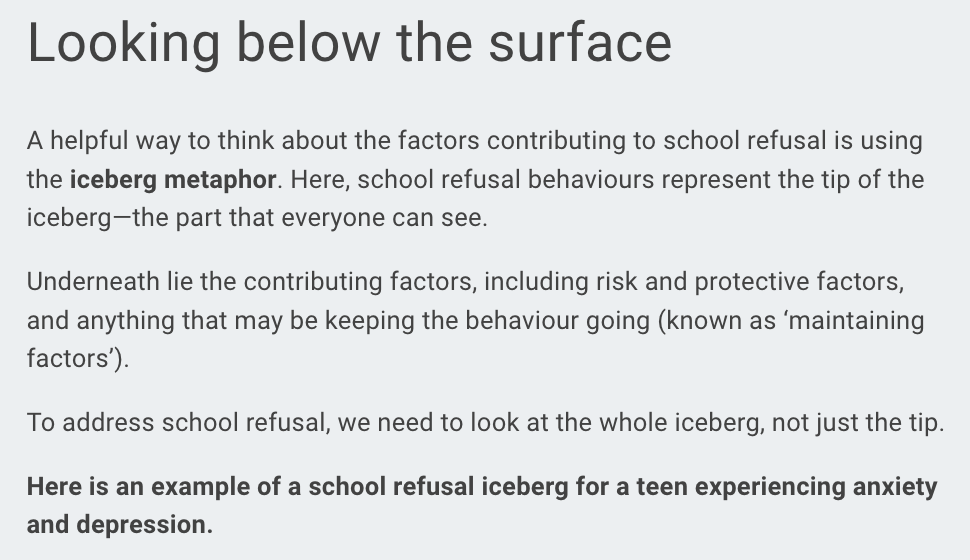  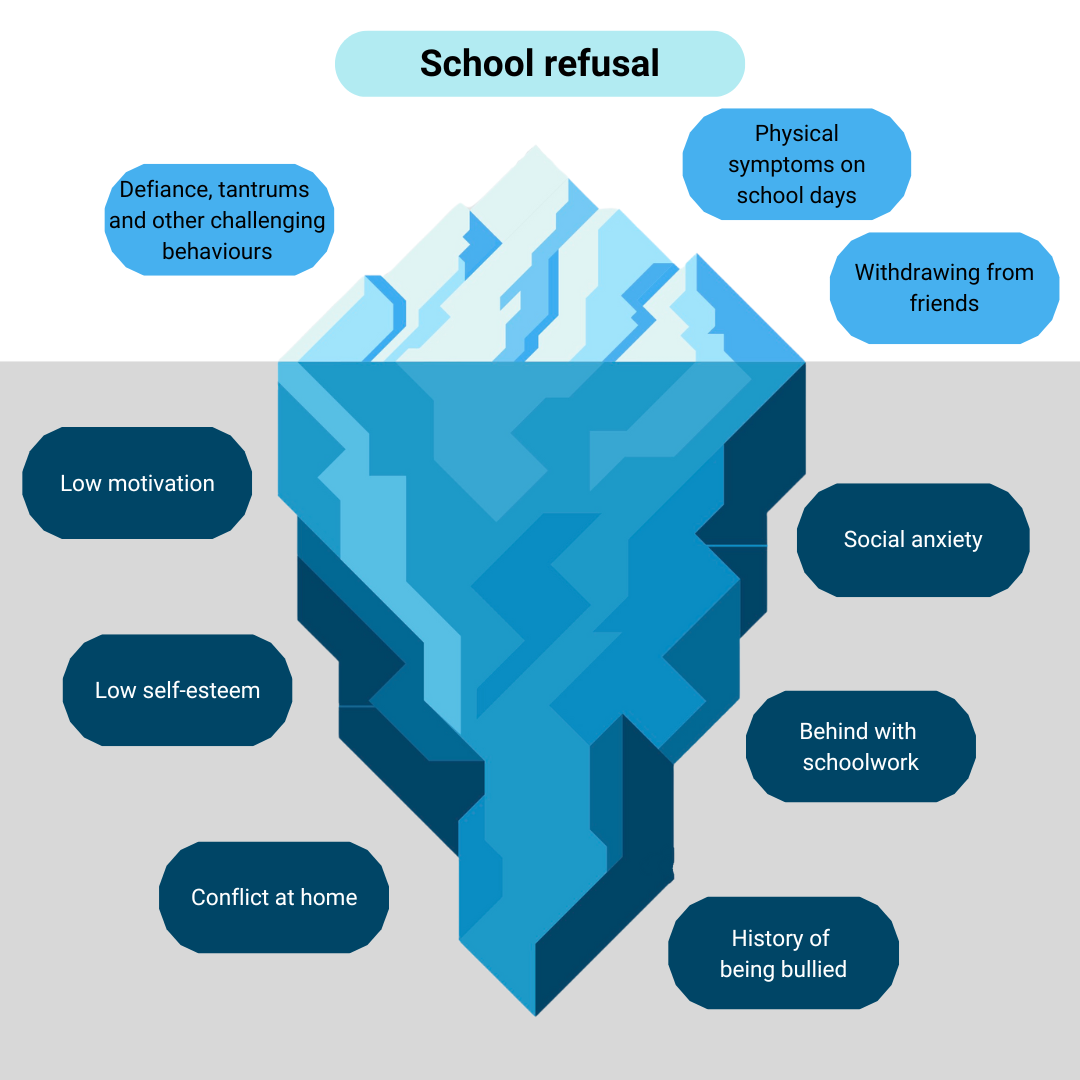 | During the corresponding coaching session, parents build an individualised school refusal ‘iceberg’ relevant to their adolescent.  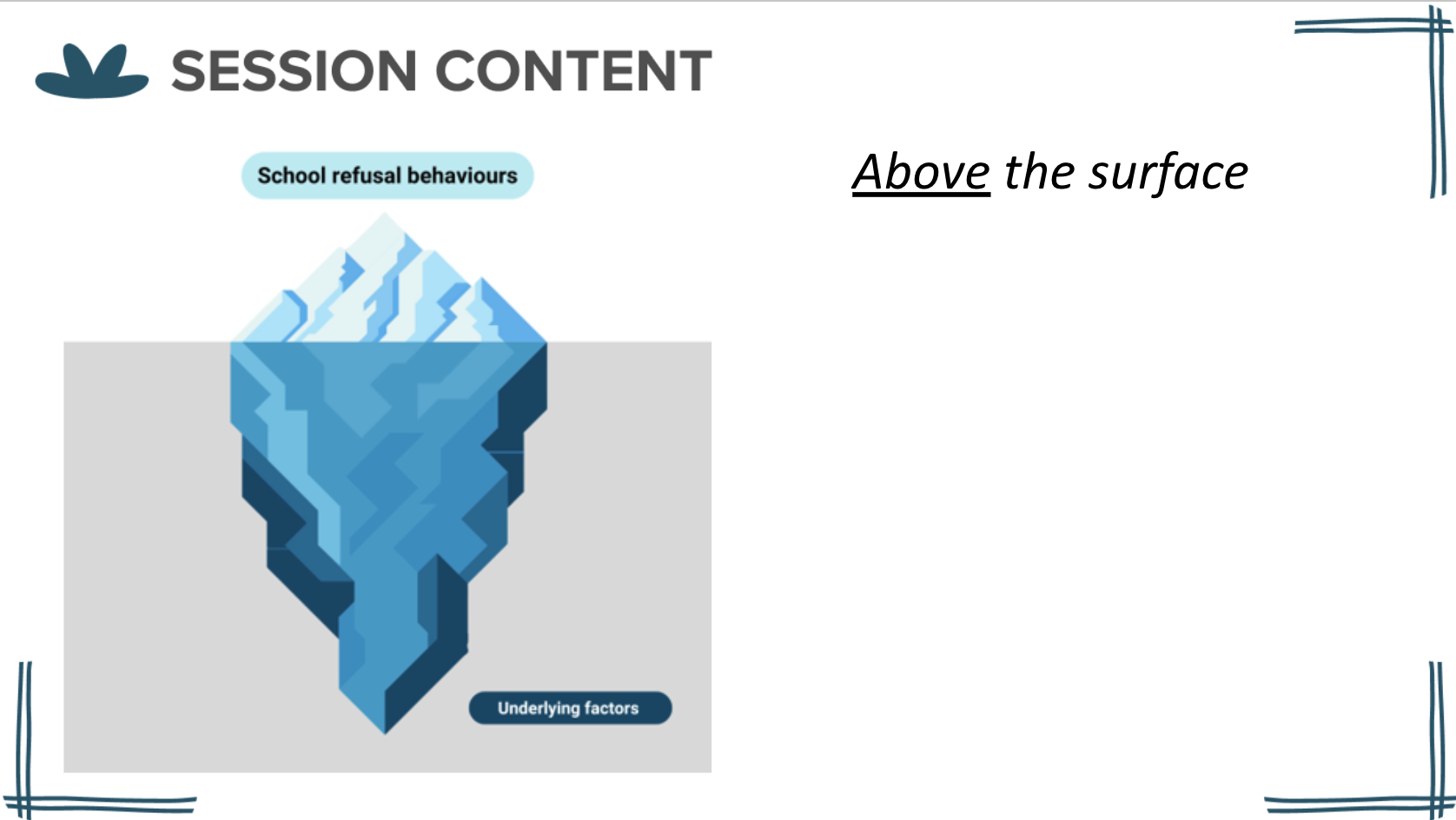  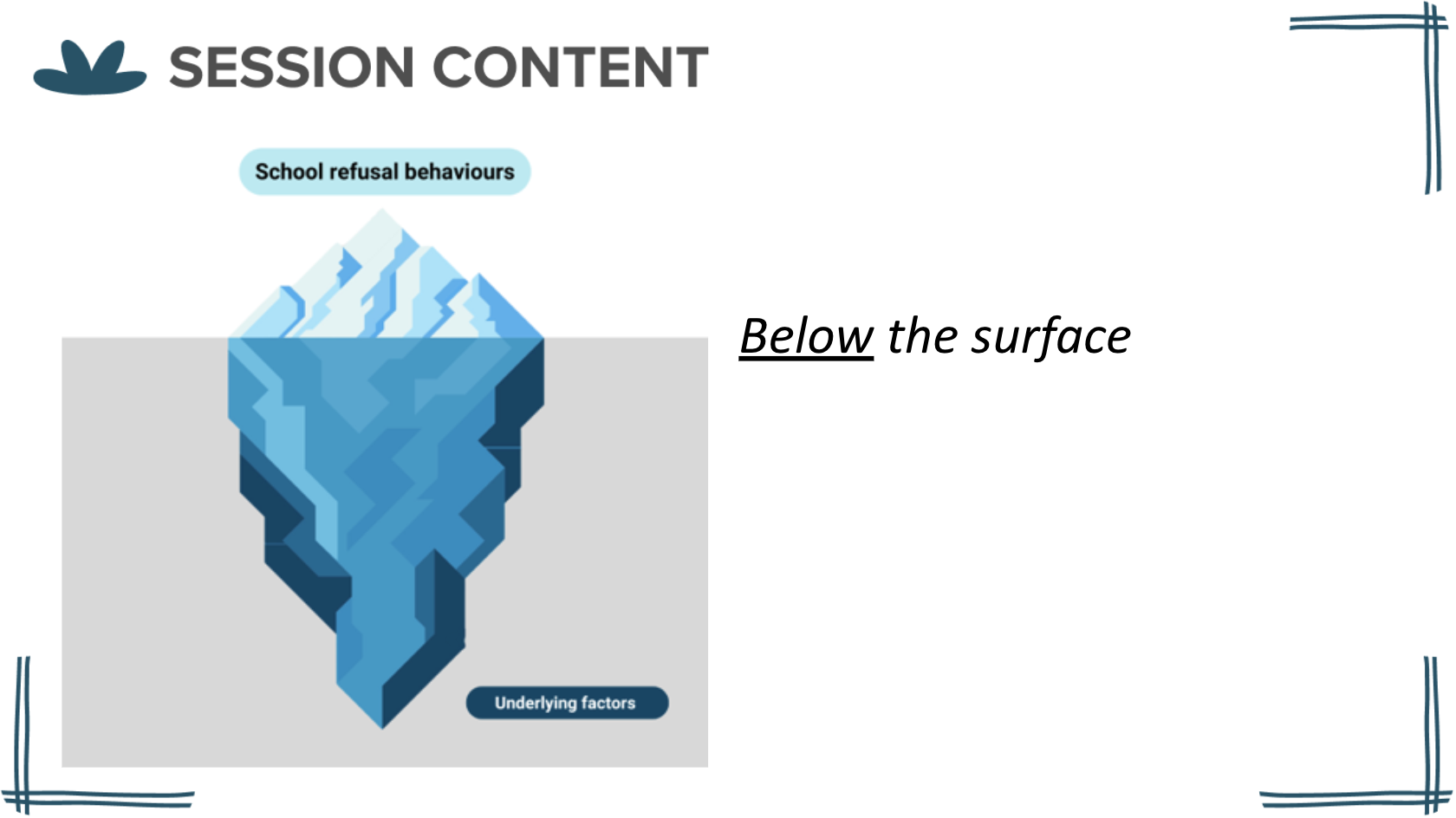 | Between sessions, parents are encouraged to reflect on and record the key points of the discussion. Where appropriate, parents are encouraged to involve their adolescent and school staff in this activity. For example, by gaining their respective inputs on the contributory factors.  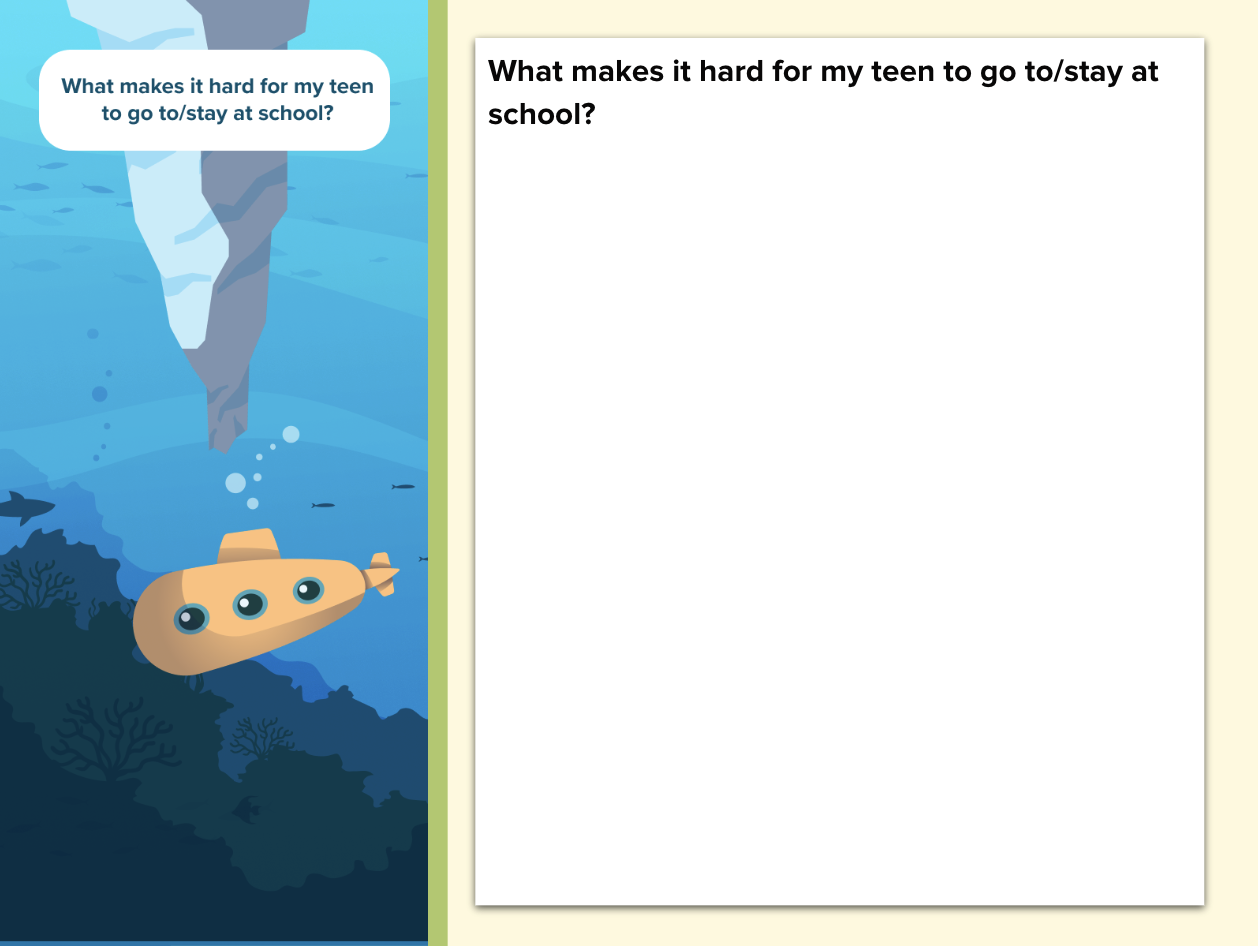 |

## **Supplement B: PiP-Ed+ Evaluation Interview Schedule**

| **Domain** | **Central Questions** | **Probes/Prompts** | **Accompanying slide/s** |
| --- | --- | --- | --- |
| Acceptability | **“To start with, it would be great to hear about your overall impressions of the program.”**  **1. Overall, what did you think of the PiP-Ed program?**  **2. Overall, did you like or dislike the PiP-Ed program?**  *Optional: After free responses, move to ‘components of PiP-Ed’ slide.*  **“You’ve mentioned some of these already, is there anything else from this list that you liked/disliked?”** | **Specific prompts:**  - [If not already covered] What, if anything, did you **like** about PiP-Ed?  - [If not already covered] What, if anything, did you **dislike** about PiP-Ed?  - How could PiP-Ed be **adapted to overcome/improve** [X]?  - Did you experience **discomfort** of any kind as a result of engaging with PiP-Ed?  - How **similar** was the PiP-Ed program to what you **expected**?  **General probes:**  -Can you tell me more about that?  -How so?  -Why / why is that / why was that the case for you?  -Why is that important to you?  -Could you give me an example? | **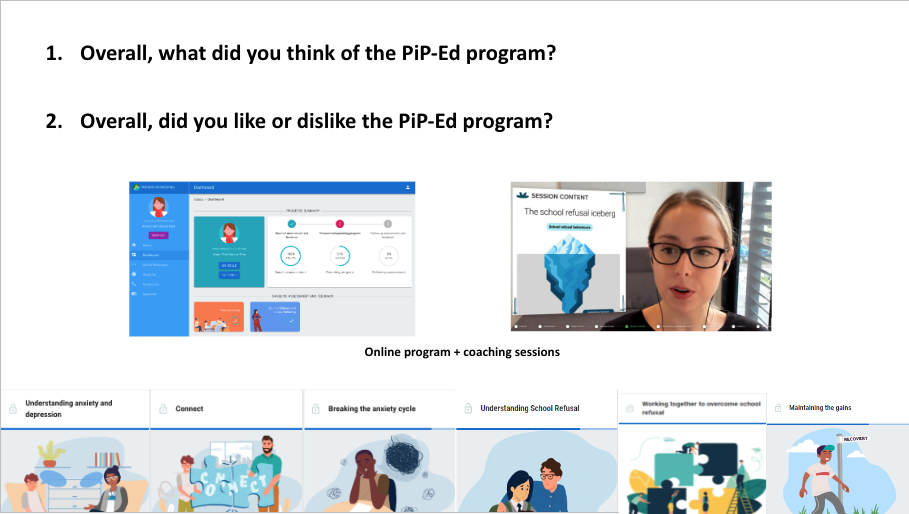**  **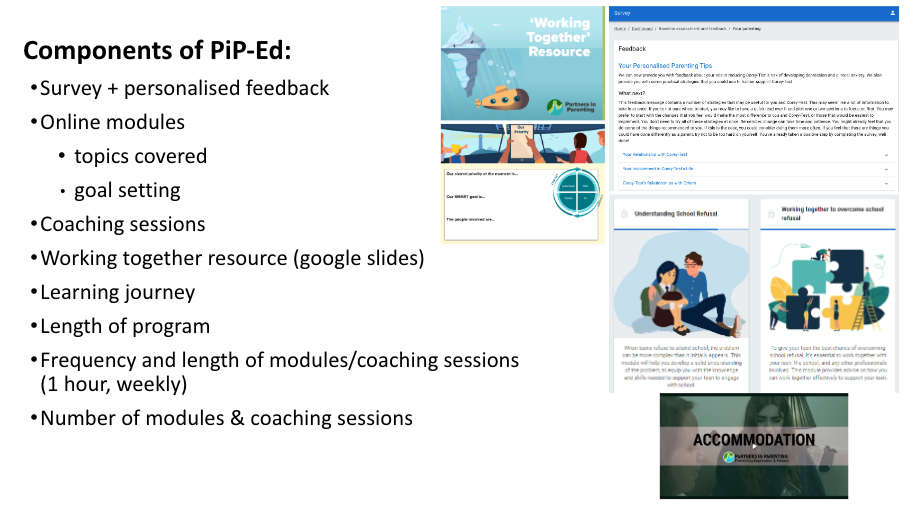** |
| Design validity | **“The program was designed to increase parents’ confidence in responding to their teen’s school refusal”.**  **3. How helpful was the program, if at all, in building your confidence to respond to [NAME]’s school refusal?** | **Specific prompts:**  - [If not already covered] Are there **situations that you feel more confident** in handling since doing the program? [If yes] Could you tell me about one of those situations?  - [For all] What **aspects of PiP-Ed contributed to your confidence** increasing in this/these way/s, and how?  [Show ‘components’ slide]: *“What, if anything, from this list contributed to your increase in confidence [besides what you already mentioned]?”*  **-** What **would you have needed from PiP-Ed for your confidence** in responding to your teen’s school refusal to increase [further]? This could be changes to existing aspects of PiP-Ed (as per the list), or any other features/components parents think would be helpful to add  **General probes:**  -Can you tell me more about that?  -How so?  -Why / why is that / why was that the case for you?  -Why is that important to you?  -Could you give me an example? | **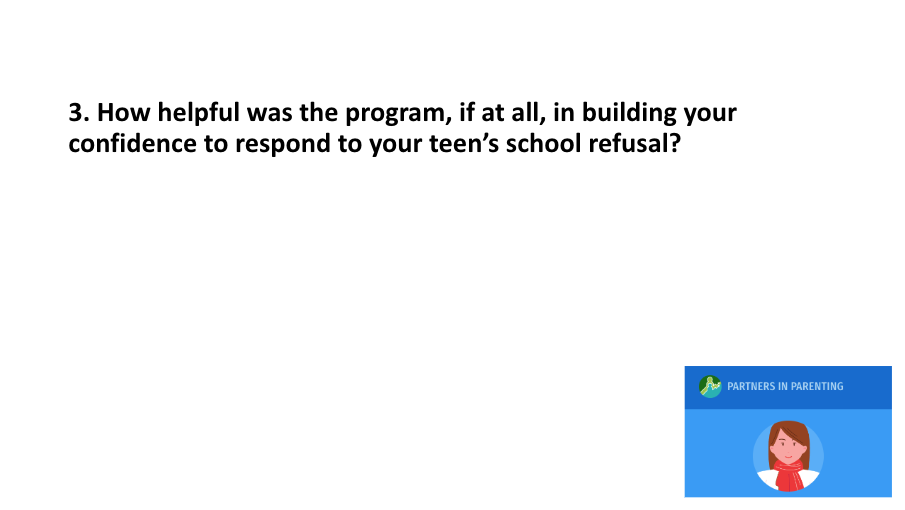**  **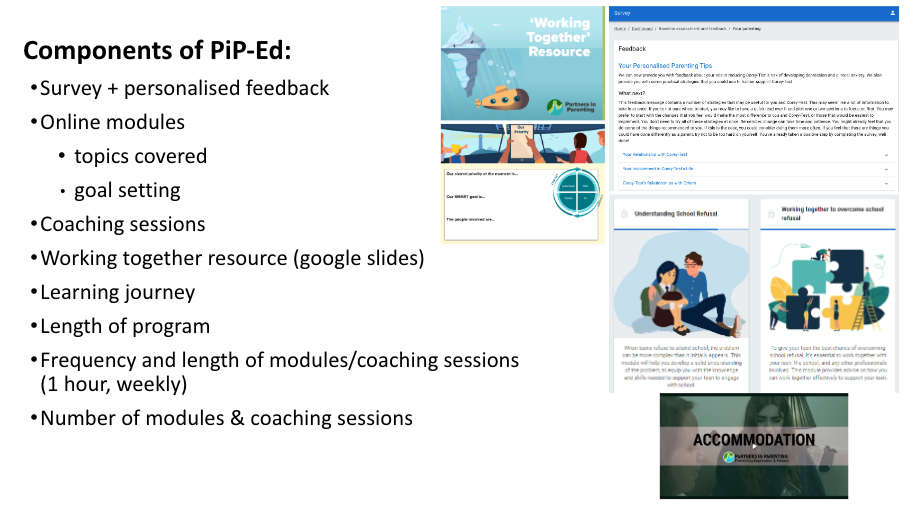** |
| Acceptability | **4. How appropriate was the program to your family context?**  **For example, who is in your family, your cultural or religious background, your financial position, your values towards parenting, education and mental health.** | **Specific prompts:**  -How well did you feel your family context was represented in the program?  - Did you feel excluded or offended by any aspects of the program?  **General probes**  -Can you tell me more about that?  -How so?  -Why / why is that / why was that the case for you?  -Why is that important to you?  -Could you give me an example? | **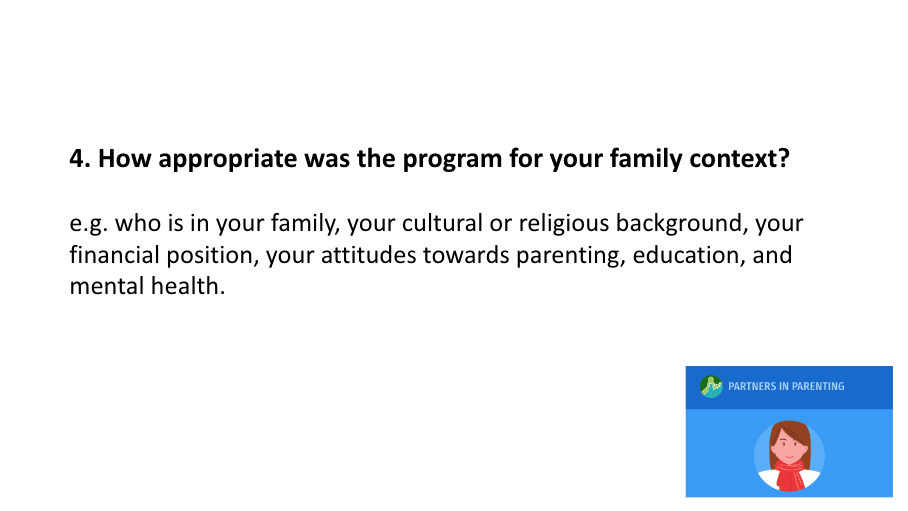** |
| Acceptability, feasibility | **5. How much effort did it take for you to do the PiP-Ed program?** | **Specific prompts:**  -How did you feel about the amount of effort required of you to do the PiP-Ed program?  **General probes**  -Can you tell me more about that?  -How so?  -Why / why is that / why was that the case for you?  -Why is that important to you?  -Could you give me an example? | **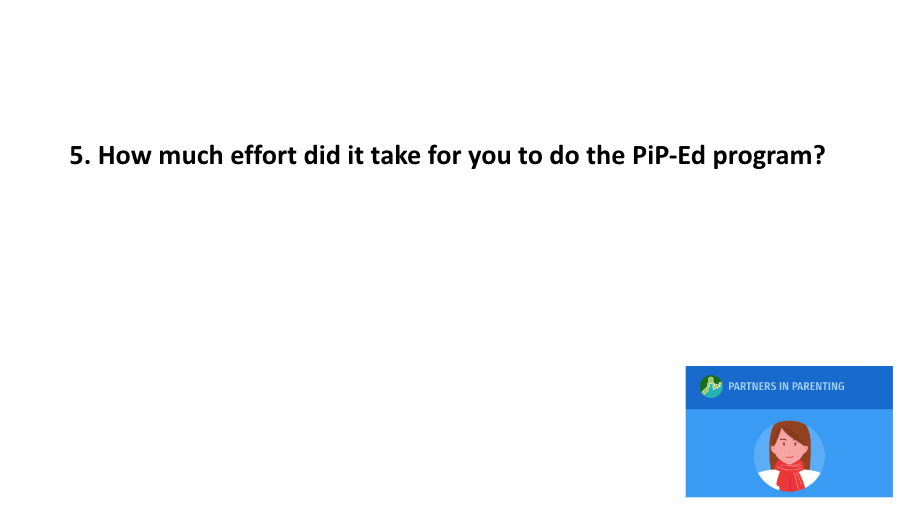** |
| Acceptability | **6. What, if anything, did you need to give up or sacrifice in order to do the PiP-Ed program?** | **Specific prompts:**  -How did you feel about having to give up/sacrifice those things in order to do the program?  **General probes**  -Can you tell me more about that?  -How so?  -Why / why is that / why was that the case for you?  -Why is that important to you?  -Could you give me an example? | **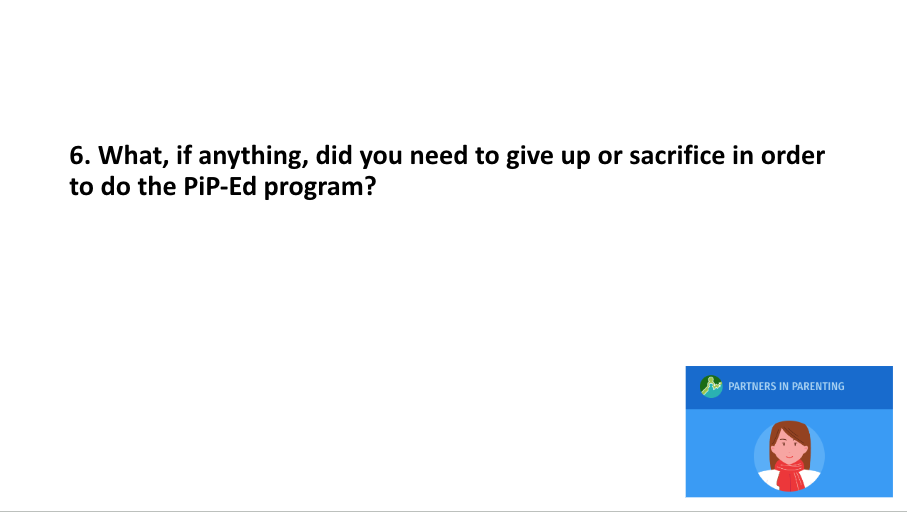** |
| Feasibility | **7. How confident did you feel about your ability to complete the PiP-Ed program?**  **(Requirements)** | **Specific probes:**  [Next slide] How confident did you feel in your ability to…  - do the online modules?  - do the weekly coaching sessions?  - complete your between-session goals?  - make adjustments to your parenting? - use the ‘working together resource’?  **General probes**  -Can you tell me more about that?  -How so?  -Why / why is that / why was that the case for you?  -Why is that important to you?  -Could you give me an example? | **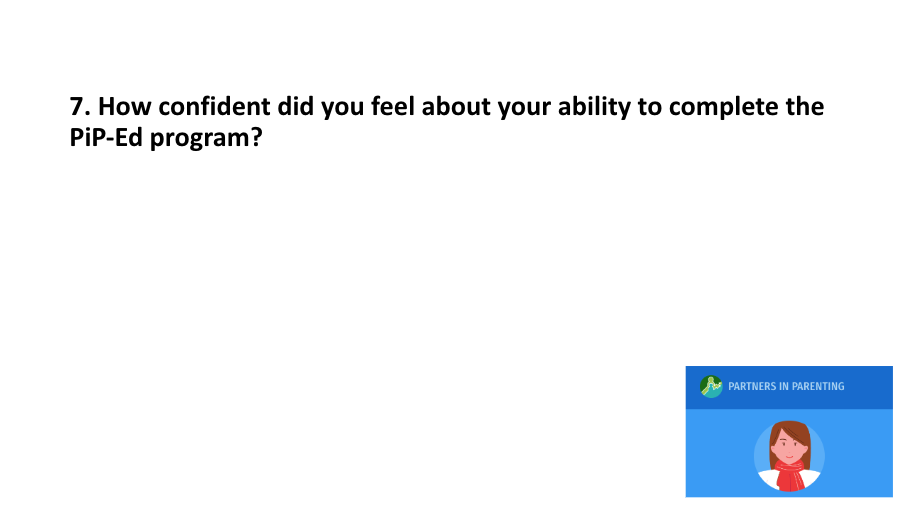**  **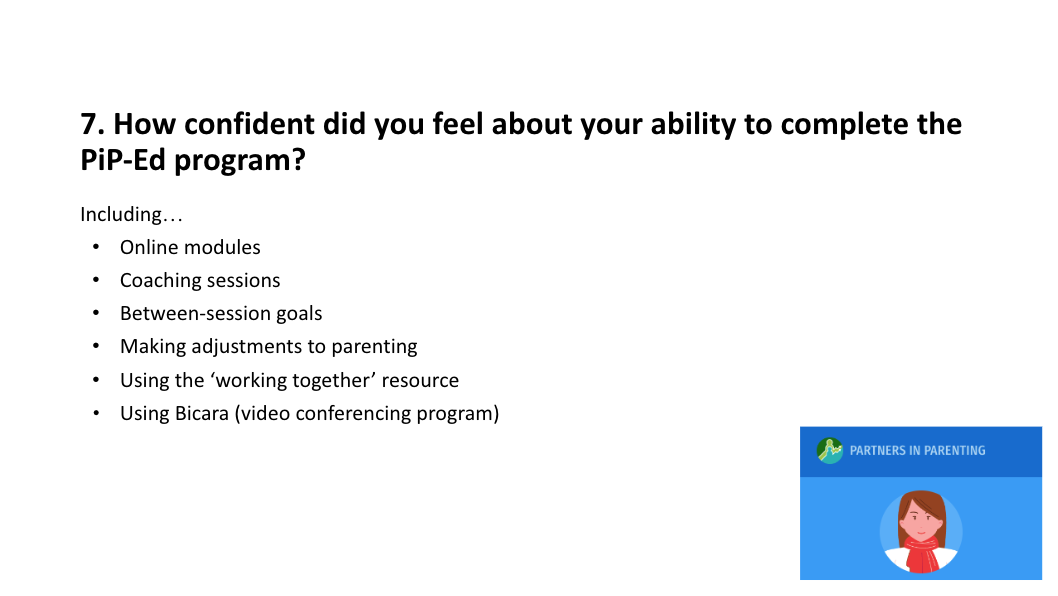** |
| Other suggestions for program improvement | **8. Do you have any other comments or suggestions for improving the program that we haven’t discussed so far?** | **General probes**  -Can you tell me more about that?  -How so?  -Why / why is that / why was that the case for you?  -Why is that important to you?  -Could you give me an example? | **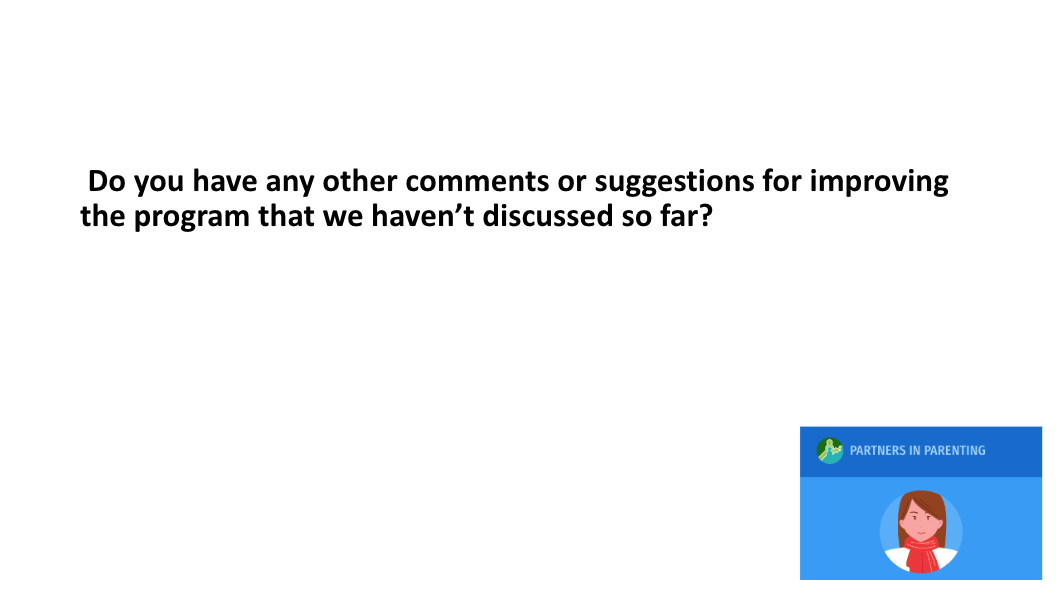** |

##

## **Supplement C: Joint Display of quantitative and qualitative findings with meta-inferences**

| **Quantitative** | | **Qualitative** |  | **Meta-inferences** |
| --- | --- | --- | --- | --- |
|  | | Themes | Sub-themes | Interpretation of mixed-methods findings |
| Acceptability of the program to end users | | | | |
| CSQ-8 score; Mean (range) | 30.73 (27-32) | Valued aspects | - Coaching sessions were the most valuable part of the program - Qualities of their coach - Content was appropriate, engaging, comprehensive, and helpful - Experiencing positive outcomes - Program instilled a positive sense of accountability - Program was worth the time and effort - Number and frequency of online and coached components | Scores on the CSQ-8 indicate the program is highly acceptable. The sub-themes confirm and add context to these scores, based on the experiences and perspectives of the interviewed parents. Practical suggestions for improving the acceptability of the program were provided. |
|  |  | Disliked aspects | - Mismatches between some content and parent circumstances, needs, or preferences - Difficult for two parents to co-participate - Issues related to video- conferencing |  |
|  |  | Suggestions for improvement | - Changes or additions to content and functionalities - Involvement of co-parents, their adolescent, and school staff |  |
| Validity of intervention design | | | | |
| Change in PSES-SR scores between baseline and post-intervention; Z (*p*) | -2.85 (*p =* .004) | Program components which increased parent confidence in responding to school refusal | - The content (evidence-based parenting strategies) - Encouragement and reassurance from their coach - Coach tailoring of goal-setting activities | Increases in PSES-SR scores between baseline and post-intervention suggest PiP-Ed+ can produce intended outcomes. Design features of the program which contributed to increases in self-efficacy scores are delineated within sub-themes, which are based on the experiences and perspectives of the interviewed parents. One key practical suggestion for improving the validity of the program design was provided. |
|  |  | Suggestions for improvement | - Provide more support to sustain progress long-term |  |
| Feasibility of end-users completing the program as intended | | | | |
| Completion rates; *n* (%)  Adherence rates; *n* (%) | Modules: 11 (79%)  Coaching sessions: 10 (71%)  Modules: 11 (79%)  Coaching sessions: 10 (71%) | Valued aspects | - Time commitment required to complete weekly activities was achievable - Program being free of charge - Flexibility of coaching sessions | Program completion and adherence rates reflect that the module-based program requirements were somewhat more feasible to complete than coaching-based requirements. The sub-themes provide some additional context based on the experiences and perspectives of interviewed parents, all of whom had completed the whole program. |
|  |  | Disliked aspects | - Some components were difficult to access or complete |  |
|  |  | Suggestions for improvement | - Changes or additions to content and functionalities |  |
| Parenting factors or circumstances which affected program acceptability, feasibility, and validity | | | | |
|  | N/A | Valued aspects | - The timeliness of the program | This qualitative theme summarises factors outside the realm of intervention design which, according to the perspectives shared during interviews, influenced the acceptability, feasibility, and validity of the program. |
|  |  | Program components which increased parent confidence in responding to school refusal | - Their own attitude and need |  |
